# The Complete Chloroplast Genome of the Vietnamese Endemic Species *Aquilaria banaense* P.H. Hô, 1986 (Thymelaeaceae): Structure, Evolution, and Phylogeny

**DOI:** 10.1002/ece3.71708

**Published:** 2025-07-27

**Authors:** Yen Thi Van, Ngoc Bao Mach, Thanh‐Thuy Duong, Thang Nam Tran, Nguyen Van Minh, Tan Duy Ngoc Nguyen, Hoang Dang Khoa Do, Thiet Minh Vu

**Affiliations:** ^1^ University of Agriculture and Forestry Hue University Hue City Vietnam; ^2^ Functional Genomic Research Center, NTT Hi‐Tech Institute Nguyen Tat Thanh University Ho Chi Minh City Vietnam; ^3^ Nguyen Tat Thanh University Center for High Technology Development Saigon Hi‐Tech Park Ho Chi Minh City Vietnam

**Keywords:** agarwood, Aquilaria, comparative genomics, genetic conservation, phylogeography

## Abstract

The genus *Aquilaria* (Thymelaeaceae) is renowned for producing agarwood, a highly valuable resinous product of significant economic and cultural value. Yet, many of its species, including the endemic *Aquilaria banaense* from Vietnam, face conservation challenges due to overexploitation. This study presents the first complete chloroplast (cp) genome of *A. banaense* to investigate its genome structure, evolutionary characteristics, and phylogenetic position. The cp genome of *A*. *banaense* was 174,810 bp, exhibiting a typical quadripartite structure with a large single‐copy (LSC) region (87,264 bp), a small single‐copy (SSC) region (3342 bp), and two inverted repeat (IR) regions (42,102 bp), encoding unique 79 protein‐coding genes, 30 tRNA genes, and four rRNA genes. Comparative analysis across *Aquilaria* species revealed conserved genomic features, including IR expansion into the SSC region, absence of *clpP1* introns, alongside moderate nucleotide divergent regions (such as *matK*‐*rps16*, *petN‐trnT‐GGU*, *rps4‐ndhJ*, and *ndhF‐rpl32*) and a distinct *accD* variant in *A. banaense*. Phylogenetic reconstruction using whole cp genomes of 13 *Aquilaria* species placed *A. banaense* sister to mainland Southeast Asian species like *A. crassna* and *A. subintegra* , clearly separated from insular Southeast Asian groups. The correlation between phylogenetic structure and geographic distribution suggests that historical biogeography and ecological factors have driven lineage divergence in *Aquilaria*. These findings highlight the genetic distinctiveness of *A. banaense* and provide valuable genetic resources for species identification and conservation planning for this vulnerable species.

## Graphical abstract

This study presents the first complete chloroplast genome of *Aquilaria banaense*, an endemic Vietnamese species, revealing its phylogenetic position within the *Aquilaria* genus and its biogeographic alignment with mainland Southeast Asia. Comparative analyses highlight conserved genome structure and unique long tandem repeat variations, emphasizing the need for expanded genomic data from Malesia to support conservation efforts for this endangered, agarwood‐producing genus.

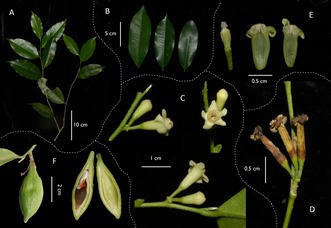

## 1 Introduction

The genus *Aquilaria*, within the Thymelaeaceae family, comprises 21 taxonomically recognized species distributed primarily across Southeast Asia, India, and southern China (Lin et al. 2023; Plant of the World Online (POWO) 2024). This genus is renowned for producing agarwood, a resinous non‐timber product with immense economic value in incense, perfumery, artisanal furniture, and traditional medicine (Hashim et al. 2016; Lee and Mohamed 2016; López‐Sampson and Page 2018; Wang et al. 2018, 2021). However, the high demand for agarwood has led to widespread overexploitation, causing significant population declines for many *Aquilaria* species. Consequently, the genus is listed under Appendix II of the Convention on International Trade in Endangered Species of Wild Fauna and Flora (CITES), with most species classified as either “Endangered” (EN) or “Vulnerable” (VU) (CITES 2005). This conservation crisis underscores the urgent need for a deeper genetic understanding of the *Aquilaria* genus, particularly its endemic taxa, which harbor unique genetic diversity and evolutionary adaptations.

**FIGURE 1:**
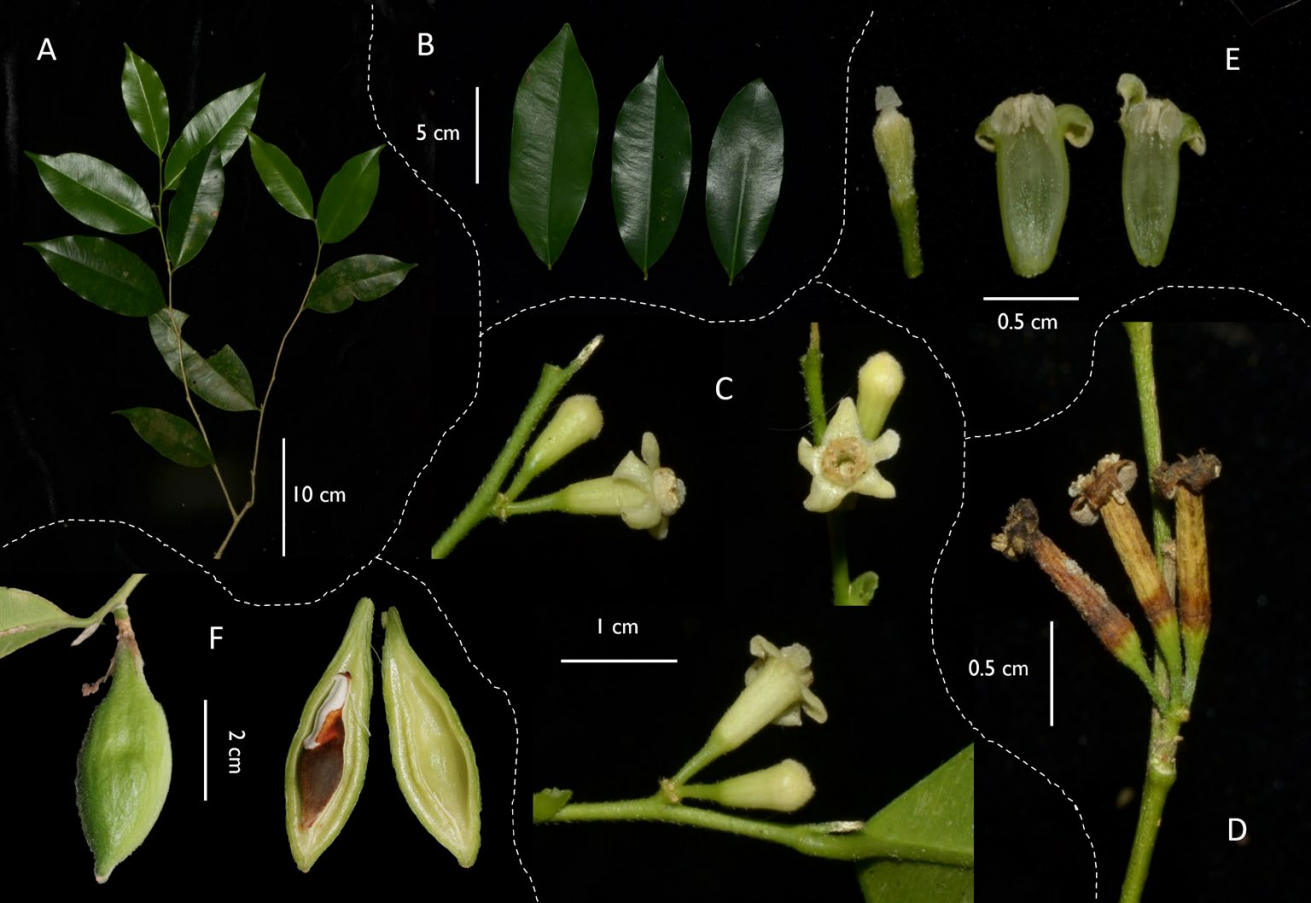
Photograph of *A. banaense* morphology. (A) Branch with compound leaves showing alternate arrangement and elliptic to oblong leaflets; (B) Leaf surfaces: The leftmost and middle leaves show the abaxial (lower) surface, which is lighter in color and duller; the right leaf shows the adaxial (upper) surface, which is darker green and glossy; (C) Flower buds and open flowers in lateral and face views; (D) Persistent floral tube after anthesis.; (E) Dissected floral parts showing stamens and ovary; (F) Immature fruit (left) and longitudinal section of fruit showing developing seed and endocarp (middle and right). The photo credit: Yen Thi Van.

Among these, *Aquilaria banaense* P.H.Hô, 1986 (synonym: *Aquilaria banaensis* ), is an endemic species restricted to the hilly terrains of Ba Na, Western Quang Nam, and Thua Thien‐Hue Provinces in Vietnam (Ho 1986; Van, Duong, et al. 2024). This dwarf tree (2–3 m tall) is distinguished by slender branches with sparse hairiness during its juvenile period (Ho 1986). Its leaves are slender, pointed at both ends, measure 5–10 cm in length and 1.6–3.5 cm in width, and lack coarse hairs. The inflorescences are axillary and typically bear three to four flowers (Figure 1). The fruit is elongated and slender, 2.5 cm long and 1.2 cm wide, with a brown shell approximately 1 mm thick containing a single seed (Ho 1986). Despite its ecological and potential economic significance, research on *A. banaense* remains limited, focusing mainly on the antimicrobial properties of phytochemicals derived from its leaves and trunks (Van, Dinh et al. 2024). Its morphological similarity to related species complicates identification, posing challenges for conservation and regulated trade. The lack of genetic data further amplifies the need for research to elucidate its biological and evolutionary profile.

Taxonomic and phylogenetic studies of *Aquilaria* within Thymelaeaceae have advanced over the past two decades, yet challenges persist. Early studies using morphological traits and molecular markers like *trnL‐trnF*, *rbcL*, and nuclear ITS classified the Thymelaeaceae into four subfamilies—Thymelaeoideae, Aquilarioideae, Gonystyloideae, and Gilgiodaphnoideae—with *Aquilaria* assigned to Aquilarioideae (Heywood 1993; van der Bank et al. 2002). However, at the lower taxonomic levels, these markers struggle to distinguish *Aquilaria* from its sister genus, *Gyrinops*, limiting their use for taxonomic studies and species authentication (Farah et al. 2018; Lee et al. 2018; Feng et al. 2019). Next‐generation sequencing (NGS) has transformed plant systematics by enabling the efficient acquisition of complete chloroplast (cp) genomes. In angiosperms, cp genomes typically range from 120 to 170 kb and encode genes involved in photosynthesis, genetic systems, and metabolic functions (Bräutigam and Gowik 2010; Straub et al. 2012; Sun et al. 2022). Their conserved structure, low mutation rates, and predominantly maternal inheritance make cp genomes highly informative for resolving evolutionary relationships, especially in morphologically complex taxa (Ruhlman and Jansen 2014; Daniell et al. 2016; Kumar et al. 2022).

To date, complete cp genomes have been reported for 12 *Aquilaria* species, supporting the monophyly of the genus and providing a framework for comparative studies (Lee et al. 2018; Hishamuddin et al. 2020). Nonetheless, additional cp genomic data are needed to resolve interspecific relationships and better understand the evolutionary history of this ecologically and economically important group. In this study, we report the first complete cp genome of *A. banaense* and conduct comparative and phylogenetic analyses with other *Aquilaria* and Thymelaeaceae species. Our findings contribute to the genomic basis necessary for *Aquilaria* conservation and agarwood research.

## 2 Materials and Methods

### 2.1 Plant Samples Collection and DNA Extraction

Fresh leaves of *A. banaense* were collected from the Sao La Nature Reserve in Hue City, Vietnam (16°28′1.10″ N 107°20′3.35″ E). A voucher specimen was deposited in the Plant Biotechnology Laboratory at the University of Agriculture and Forestry, Hue University, Vietnam (accession number HUAF_BA_4, contact person Thuy Duong Thanh, duongthanhthuy@huaf.edu.vn). Sample collection for scientific study was conducted with permission granted by the Management Board of Sao La Conservation Area, Hue City. Genomic DNA was extracted from the fresh leaves using the cetyltrimethylammonium bromide (CTAB) method (Doyle and Doyle 1987; Porebski et al. 1997). DNA concentration and purity were assessed using a Nanodrop spectrophotometer (Thermo Fisher Scientific, USA) and 1% agarose gel electrophoresis. High‐quality DNA samples (concentrations ≥ 2.00 ng μL^−1^, OD_260/280_ ratio between 1.8 and 2.0) were used for library preparation and sequencing.

### 2.2 Chloroplast Genome Sequencing and Annotation

A paired‐end DNA library (2 × 150 bp) was prepared and sequenced on the Illumina Nextseq platform at KTest Science Ltd. Company (Ho Chi Minh City, Vietnam). Raw reads were used for *de novo* assembly by GetOrganelle software v1.7.7.0, using its embryophyta plastome (embplant_pt) database, to construct a complete circular cp genome sequence (Jin et al. 2020). Sequencing depth and coverage of the assembled genome were plotted using Python scripts (Ni et al. 2023). The genome was annotated by using GeSeq‐CHLOROBOX, with parameters optimized for angiosperm cp genome (Tillich et al. 2017). A similarity cutoff for the translated BLAST search was 25% for protein and 85% for rRNA and tRNA genes. The annotation result was further validated using tRNAscan‐SE v2.0 (Chan and Lowe 2019) and BLAST+ (Camacho et al. 2009). The annotated genome map was sketched using OrganellarGenomeDRAW (OGDRAW) v1.3.1 (Greiner et al. 2019).

### 2.3 Analysis of IR Region Dynamics

The expansion and contraction of the IR regions were assessed by characterizing the junction sites among four cp genome components (i.e., LSC, SSC, and two IR regions) and their adjacent gene arrangement. A schematic diagram of adjoining sites was generated using CPJSdraw v.0.0.1 (Li et al. 2023).

### 2.4 Comparative Genome Analysis and Nucleotide Divergence

Genome structural variation was evaluated through global and pairwise alignments. Locally collinear blocks (LCBs) in investigated cp genomes were identified using progressiveMauve alignment (Darling et al. 2010) implemented in Geneious Prime software version 2024.0.7 (Geneious 2024). Match seed weight and minimum LCB score were computed automatically. Global full alignment ran with the “Assumed collinear genomes” setting, and the results were displayed using MAUVE (Darling et al. 2004). Pairwise genome comparisons were performed using the mVISTA program with the Shuffle‐LAGAN* glocal framework alignment algorithm (Mayor et al. 2000; Frazer et al. 2004). The RankVISTA probability threshold (*p*) was set to 0.5 to refine the sensitivity of detected variations.

Nucleotide diversity (Pi) within the *Aquilaria* cp genomes was quantified by DnaSP v6 (Rozas et al. 2017). A single copy of the IR regions was removed to eliminate redundancy. Sequences were aligned using the Mauve whole genome alignment in Genious Prime. Aligned sequences in Nexus format (.nex) were used as input with a sliding window length of 2000 nucleotides and a step size of 100 nucleotides.

### 2.5 Identification of Long Tandem Repeats and Codon Usage Analysis

Long repetitive repeats (LTRs) were characterized using the REPuter web server (https://bibiserv.cebitec.uni‐bielefeld.de/reputer) (October 15th, 2024) (Kurtz et al. 2001). Parameters included a Hamming distance of three, a minimum repeat length of 30 bp, and a maximum of 200 sequences with the best E‐value. Repeat types analyzed included forward, reverse, complementary, and palindromic repeats. The repeat profiles of each cp genome were summarized and compared.

Relative synonymous codon usage (RSCU) indices of the PCGs in the *A. banaense* cp genome were calculated using CodonW v1.4.4 (Peden 1999) and visualized by RStudio software v2024.09.0 + 375 (RStudioTeam 2020) with ggplot2, tidyverse, patchwork, magrittr, and dplyr packages.

### 2.6 Phylogenetic Analysis

Phylogenetic relationships were inferred using cp genome sequences from 14 species of the Thymelaeoideae subfamily, including 12 *Aquilaria* species, the newly sequenced *A. banaense*, and one outgroup, *Daphne championii*. The cp genome sequences were retrieved from GenBank (Table S1).

The alignment of whole cp genomes was used to determine the optimal evolutionary model in jModelTest v2.1.10 (Darriba et al. 2012). Phylogenetic reconstruction was performed using Bayesian inference (BI) and maximum likelihood (ML) methods. For BI, the analyses were carried out using MrBayes v3.2 (Ronquist et al. 2012) with the following parameters: 1,000,000 generations, a diagnosis frequency of 5000, and a burn‐in fraction of 0.25. The run stopped when the standard deviation of split frequency fell below 0.01. For ML estimation, the W‐IQ‐TREE web server (Trifinopoulos et al. 2016) was employed utilizing ultrafast bootstrap analysis with 1000 bootstrap replicates, the ML‐optimized state frequency, and default settings for other parameters. The resulting phylogenetic tree, combining the outputs from both methods, was visualized using FigTree v1.4.4 (Rambaut 2018).

## 3 Results

### 3.1 Characteristics of *Aquilaria banaense* Cp Genome

**FIGURE 2:**
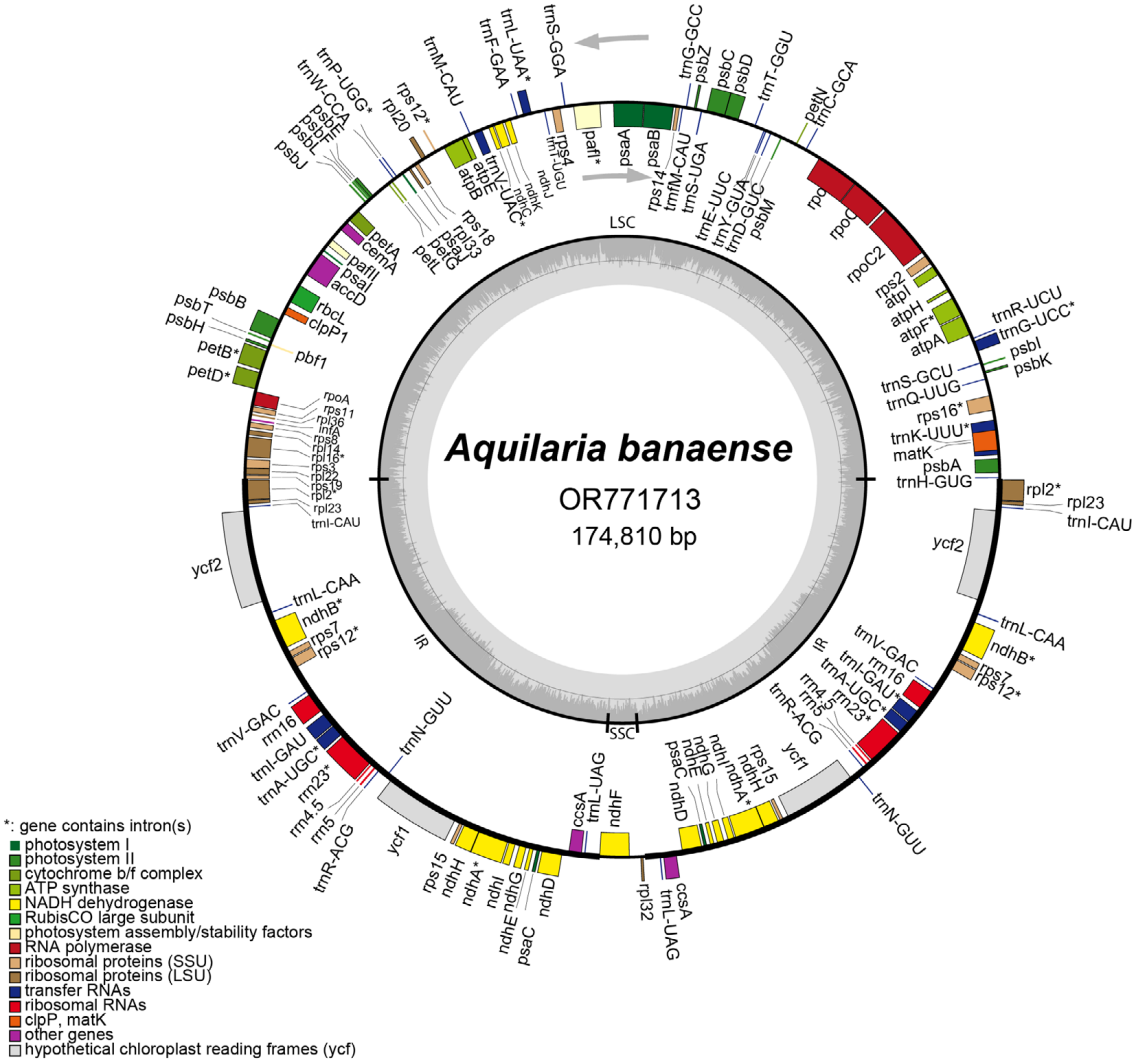
Circular chloroplast genome map of *A. banaense*. The blocks represent genes and are colored by their functions. Genes located outside and inside the circle are transcribed in counterclockwise and clockwise directions, respectively. The inner circle delineates four regions: Large Single Copy (LSC), Small Single Copy (SSC), and Inverted Repeat regions (IRA and IRB). A gray histogram in the inner circle illustrates GC content variation across the cp genome.

A total of 24,573,182 clean paired‐end reads were obtained to assemble the *A. banaense* cp genome. The final assembly was 174,810 bp in length, with an average sequencing depth of 896.25× and a minimum depth of 362× (Figure S1), indicating robust data quality for downstream analyses. The *A. banaense* cp genome exhibited a typical quadripartite structure found in most land plants, comprising a large single‐copy (LSC) region of 87,264 bp, a small single‐copy (SSC) region of 3342 bp, and two inverted repeat (IR) regions of 42,102 bp each (Figure 2). Its overall GC content was 36.7%.

Annotation of the *A. banaense* cp genome identified 140 genes, including 95 protein‐coding genes (PCGs), 38 tRNA genes, and eight rRNA genes (Table 1). A notable feature is the expansion of the IR regions into the SSC region, resulting in the duplication of 16 PCGs, eight tRNA genes, and four rRNA genes. Consequently, the SSC region was reduced to 3342 bp and retained only the *ndhF* and *rpl32* genes. Of the annotated genes, 15 PCGs and eight tRNA genes contained introns, with most harboring a single intron. Exceptions included the *pafI* and *rps12* genes, each with two introns, while the *clpP1* gene lacked any introns. The largest intron (2526 bp) was found within *trnK*. Additionally, a termination codon leading to a premature stop in the *infA* gene was identified at the nucleotide positions 82,905‐82,908. The *rps12* gene was determined to be *trans‐*splicing. The gene structure, including *cis‐* and *trans‐*splicing genes, was generated by the CPGView tool (Figure S2) (Liu et al. 2023).

**TABLE 1:** List of annotated genes in the *A. banaense* chloroplast genome.

Gene types	Functional group	Gene name
95 Protein‐coding Genes (PCGs)	Photosystem I factor	*pafI* ^*2*^, *pafII, psaA, psaB, psaC (x2), psaI, psaJ, pbf1*
	Photosystem II factor	*psbA, psbB, psbC, psbD, psbE, psbF, psbH, psbI, psbJ, psbK, psbL, psbM, psbT, psbZ*
	Cytochrome	*petA, petB* ^*1*^, *petD* ^*1*^, *petG, petL, petN*
	ATP synthases	*atpA, atpB, atpE, atpF* ^*1*^, *atpH, atpI*
	Large unit of Rubisco	*rbcL*
	NADH dehydrogenase	*ndhA* ^*1*^ *(x2), ndhB* ^*1*^ *(x2), ndhC, ndhD (x2), ndhE (x2), ndhF, ndhG (x2), ndhH (x2), ndhI (x2), ndhJ, ndhK*
	ATP‐dependent protease subunit P	*clpP*
	Envelope membrane protein	*cemA*
	Large units of ribosome	*rpl2* ^*1*^ *(x2), rpl14, rpl16* ^*1*^, *rpl20, rpl22, rpl23 (x2), rpl33, rpl36*
	Small units of ribosome	*rps2, rps3, rps4, rps7 (x2), rps8, rps11, rps12* ^*2*^ *(x2), rps14, rps15 (x2), rps16* ^*1*^, *rps18, rps19*
	RNA polymerase	*rpoA, rpoB, rpoC1* ^*1*^, *rpoC2*
	Initiation factor	infA*
	Miscellaneous protein	*accD, ccsA (x2), matK*
	Hypothetical proteins and conserved reading frames	*ycf1 (x2), ycf2 (x2)*
38 transfer RNA (tRNAs)	*trnA‐UGC* ^*1*^ *(x2), trnC‐GCA, trnD‐GUC, trnI‐GAU* ^*1*^ *(x2), trnE‐UUC* ^*1*^, *trnF‐GAA, trnG‐GCC, trnG‐UCC* ^*1*^, *trnH‐GUG, trnK‐UUU* ^*1*^, *trnL‐CAA (x2), trnL‐UAA* ^*1*^, *trnL‐UAG (x2), trnM‐CAU, trnI‐CAU (x2), trnfM‐CAU, trnN‐GUU (x2), trnP‐UGG, trnQ‐UUG, trnR‐ACG (x2), trnR‐UCU, trnS‐GCU, trnS‐GGA, trnS‐UGA, trnT‐GGU, trnT‐UGU, trnV‐GAC (x2), trnV‐UAC* ^*1*^, *trnW‐CCA, trnY‐GUA*	
8 Ribosomal RNA (rRNAs)	*rrn16S (x2), rrn23S (x2), rrn4.5S (x2), rrn5S (x2)*	

*Note:* ^1^Genes have one intron; ^2^Genes have more than one intron; *Terminated by a stop codon in the middle of the sequence; *x2*: genes that duplicated in IR regions.

### 3.2 Long Tandem Repeat Analysis

**FIGURE 3:**
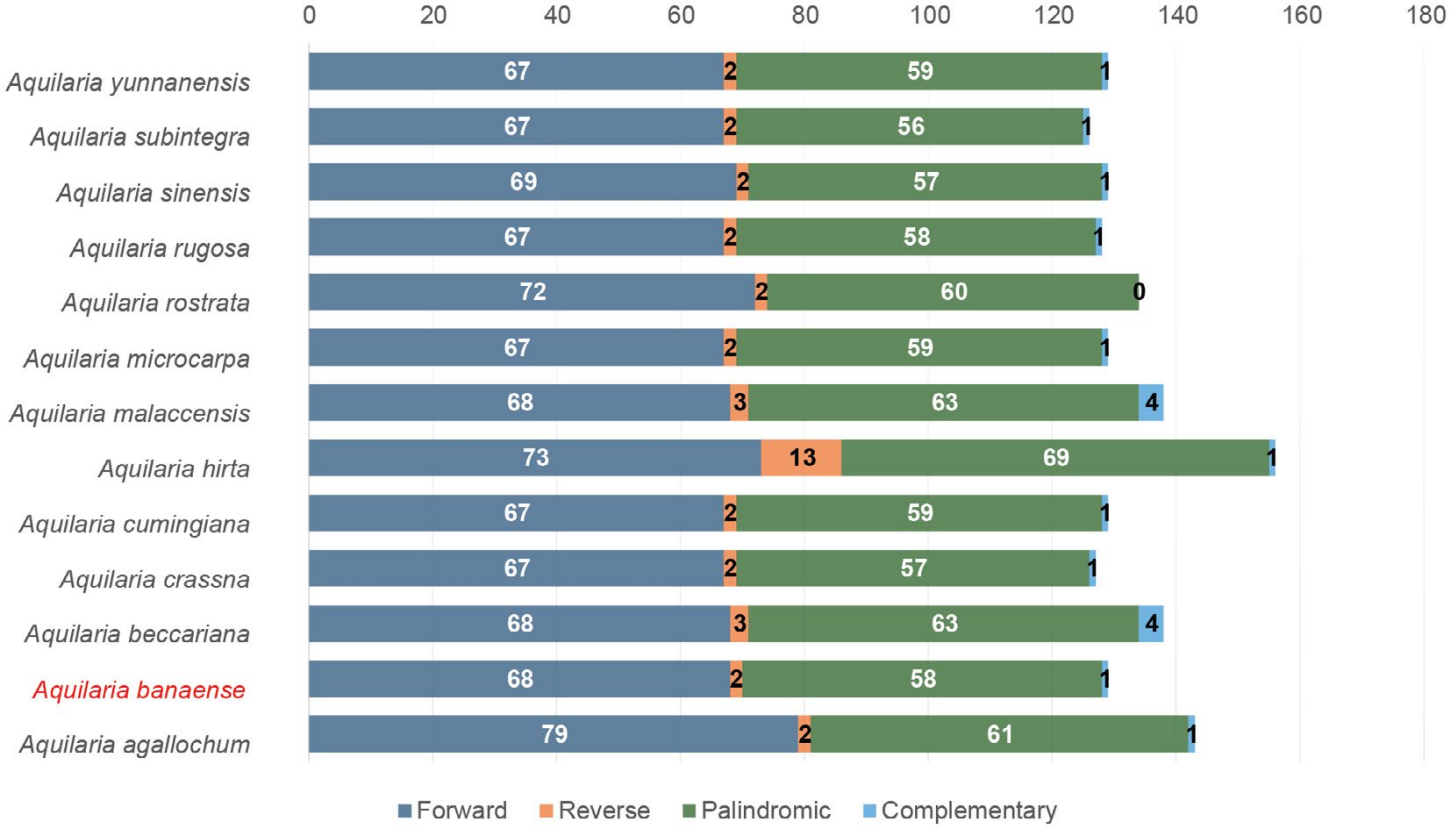
Distribution of long tandem repeats in the cp genomes of 13 *Aquilaria* species. The figure summarizes repeat types and counts across species.

We analyzed cp LTRs in 13 *Aquilaria* species (Figure 3). Forward repeats (67–79 copies) and palindromic repeats (56–63 copies) were the most abundant (Figure 3). Notably, *A. hirta* showed an outlier with 69 palindromic repeats. Reverse repeats were less common, typically found in two copies, except *A. hirta* (13 copies), *A. beccariana* (3 copies), and *A. malaccensis* (3 copies). Complementary repeats were rare, with one copy in 11 species, none in *A. rostrata* , and four in *A. beccariana* and *A. malaccensis* . Interestingly, *A. yunnanensis* , *A. cumingiana* , and *A. microcarpa* shared identical LTR counts and proportions across all repeat types, hinting at close evolutionary relationships among these species. These findings highlight the conserved yet variable nature of LTRs in *Aquilaria*, with species‐specific differences potentially linked to unique evolutionary pressures or adaptations.

### 3.3 Comparative Chloroplast Genome Analysis

**FIGURE 4:**
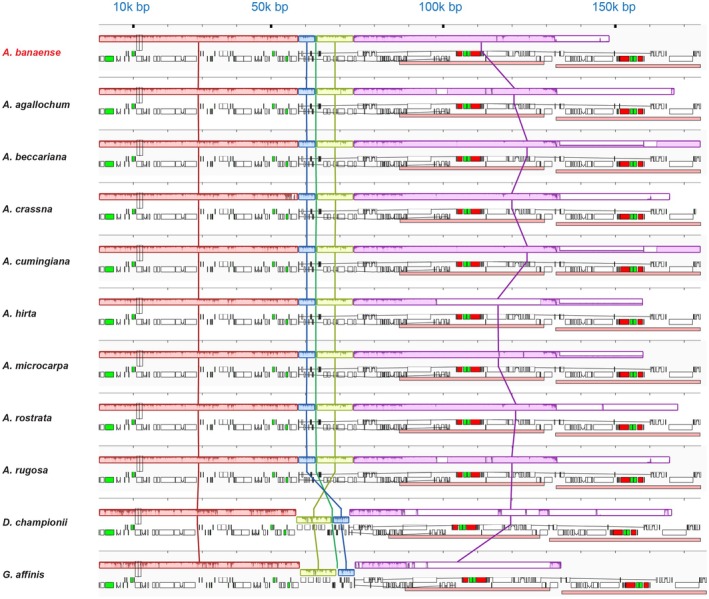
MAUVE alignment of 11 Thymelaeaceae cp genomes. Five LCBs are colored blocks connected by vertical lines indicating their positions. Blocks above the horizontal lines are in forward orientation, while those below indicate inversion. White, green, and red boxes represent PGCs, tRNAs, and rRNAs, respectively; pink boxes denote Inverted Repeat regions. Unaligned regions appear as white regions within the LCB. A genome sequence similarity profile is delineated inside the blocks. The height of the similarity profile represents the average level of conservation in that area of the genomic sequence and is inversely proportional to the average entropy of alignment columns within a specified region of the alignment. Areas outside the blocks exhibit no detectable homology among the input genomes.

To explore structural variations and conserved regions, we compared the *A. banaense* cp genome with other *Aquilaria* species and related members of the Thymelaeaceae family. Mauve alignment showed high synteny and gene order consistency among *Aquilaria* species, with five LCBs showing large consistency in sequence and organization (Figure 4). The only variation was a slight shift in the LCB position observed within the IRB. Unlike *Aquilaria*, *Daphne championii* (GenBank ID NC_068716) and *Gonystylus affinis* (GenBank ID NC_052860) exhibited LCB inversions in the LSC region (Figure 4).

**FIGURE 5:**
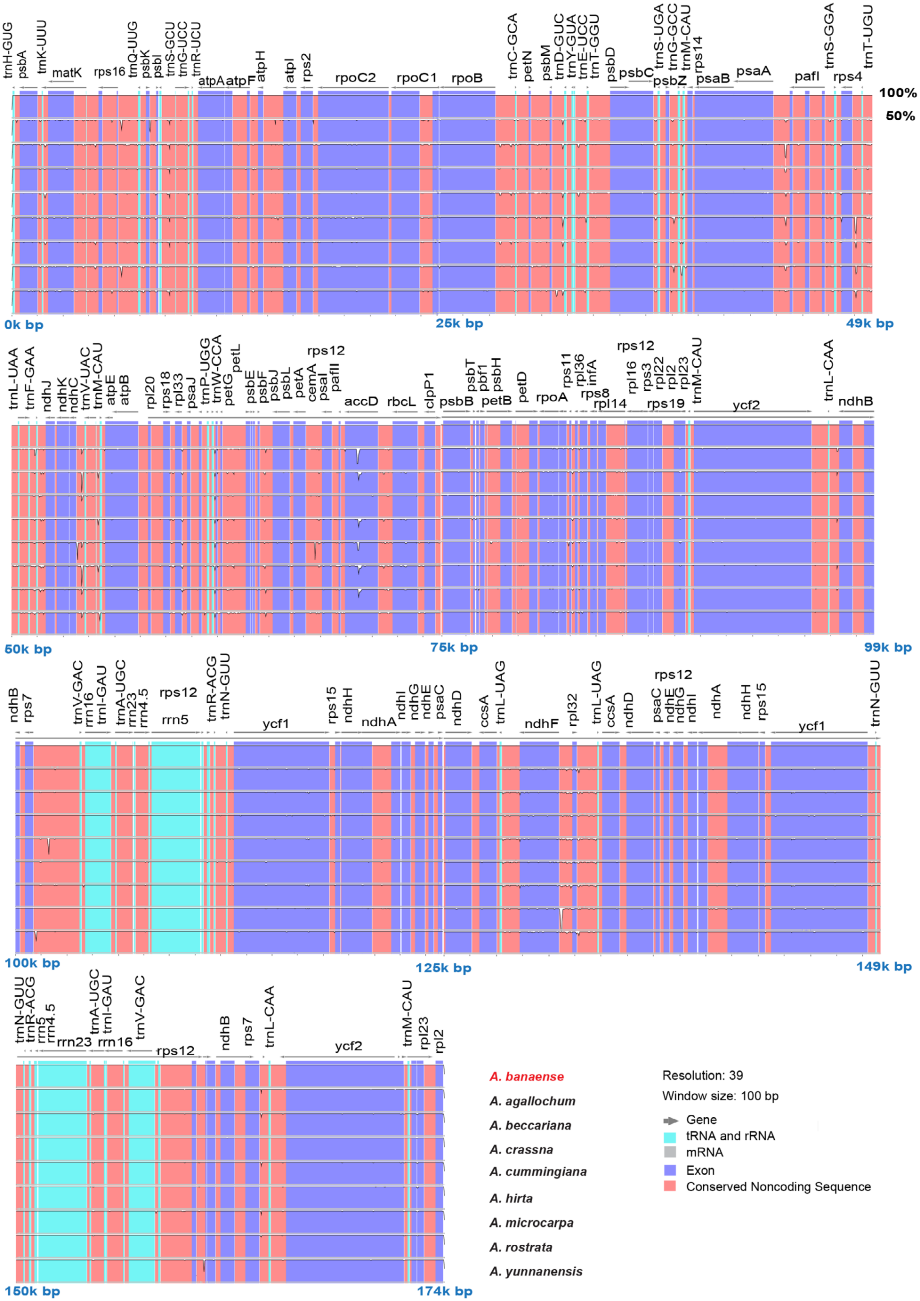
Pairwise cp genome alignment of *A. banaense* versus other *Aquilaria* species using mVISTA. The graph shows the percentage of similarity (50–100%, y‐axis) relative to the reference sequence, with a window size of 100 bp and a step size of 39 bp.

Pairwise mVISTA comparisons further confirmed a high degree of similarity among the cp genomes of *A. banaense* and other *Aquilaria* species (Figure 5). The highest divergence was observed in the *accD* region, consistent with previous studies highlighting this gene as a hotspot for variability in angiosperm cp genomes (Gurdon and Maliga 2014; Li et al. 2018; Nováková et al. 2019; Claude et al. 2022). At the whole‐genome level, *A. agallochum* exhibited the greatest divergence from *A. banaense*. Notably, no rearrangements or inversions were detected across the *Aquilaria* cp genomes, further emphasizing their structural uniformity.

### 3.4 Dynamics of Inverted Repeat Regions

**FIGURE 6:**
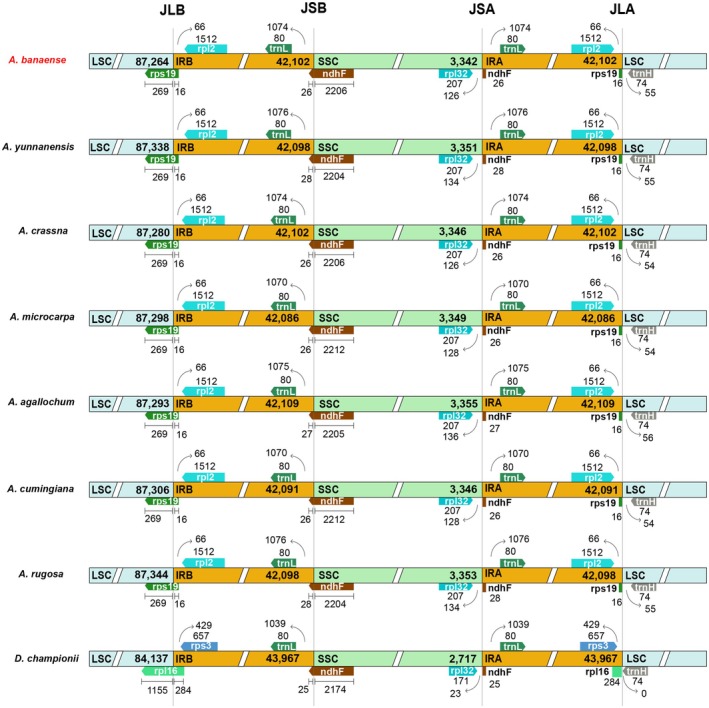
Comparison of single‐copy (SC)/Inverted Repeats (IR) in Thymelaeaceae species. Genes are not drawn to scale. Slashes (//) mean the blocks displayed only the starts and ends of the regions. Junction sites include JLB (LSC/IRB), JSB (IRB/SSC), JSA (SSC/IRA), and JLA (IRA/LSC). Numbers indicate the length of genes and regions.

The boundaries of IR regions in the cp genomes of *A. banaense* and seven other *Aquilaria* species were analyzed (Figure 6). The results revealed uniform junction sites between LSC, SSC, and the two IR regions. Specifically, the LSC/IRB boundaries (JLB) were consistently located within the *rps19* gene, while the LSC/IRA junctions (JLA) were found between the *trnH* and *rpl12* genes. The SSC/IRB boundaries (JSB) were positioned within the *ndhF* gene, and the SSC/IRA junctions (JSA) were located between the *rpl32* and *trnL* genes. These uniform boundaries suggest a highly conserved IR structure within the *Aquilaria*. In comparison, other genera within the Thymelaeaceae family exhibit distinct IR boundary arrangements. For example, in *Daphne championii*, the JLB was found within the *rps16* gene, and the JLA boundaries were between the other copies of *rps16* and *trnH*. These structural differences underscore the unique evolutionary trajectory of IR regions in *Aquilaria* compared to other taxa in the same family.

### 3.5 Nucleotide Divergence in *Aquilaria* and Codon Usage in *A. banaense* Cp Genomes

**FIGURE 7:**
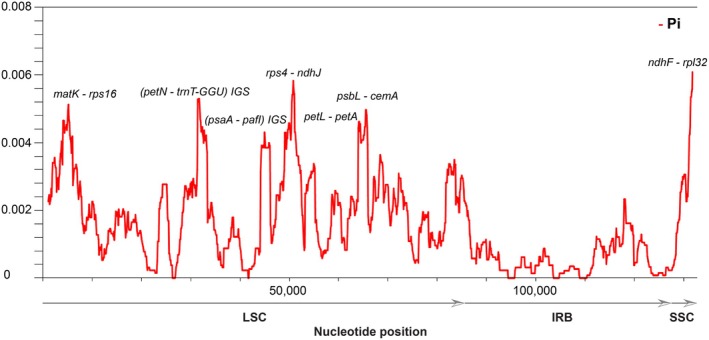
Nucleotide divergence in chloroplast genomes of 13 *Aquilaria* species. The y‐axis represents the Pi value; the x‐axis indicates the nucleotide position. The sliding window length and step sizes were 2000 and 100 bp, respectively. Arrows show the direction of regions. The cut‐off value was set at 0.004, and peaks with respective Pi values greater than the cut‐off were annotated.

To assess the pattern of nucleotide variation, the complete cp genomes of 13 *Aquilaria* species were analyzed. Overall, noncoding regions exhibited higher levels of divergence than coding regions, consistent with weaker selective constraints on intergenic spacers (Figure 7). Among the four structural regions, the SSC region displayed the highest nucleotide divergence (average Pi = 0.004), followed by the LSC region (average Pi = 0.002), while the two IR regions remained highly conserved (average Pi = 0.0006). Within the LSC region, highly divergent regions included *matK*‐*rps16*, *rps4*‐*ndhJ*, and *psbL‐cemA*, each with Pi values exceeding 0.004. The SSC region, despite being the smallest, harbored the most variable site at the *ndhF‐rpl32* intergenic spacer, with a Pi value of 0.006.

Several intergenic regions were identified as divergent hotspots, including *petN‐trnT‐GGU*, *rps4‐ndhJ*, *matK‐rps16*, and *ndhF‐rpl32*, with average Pi values greater than 0.005. The findings are consistent with previous studies on cp genomes of *Aquilaria* and other Thymelaeaceae members, which have also emphasized the variability of intergenic spacers, particularly *matK‐rps16*, *petA‐cemA*, and *ndhF*‐*rpl32* (Hishamuddin et al. 2020; Kim et al. 2021; Qian et al. 2021). Overall, this study reinforces the utility of intergenic regions for phylogenetic and evolutionary studies within the *Aquilaria* and highlights the conserved nature of the IR regions.

**FIGURE 8:**
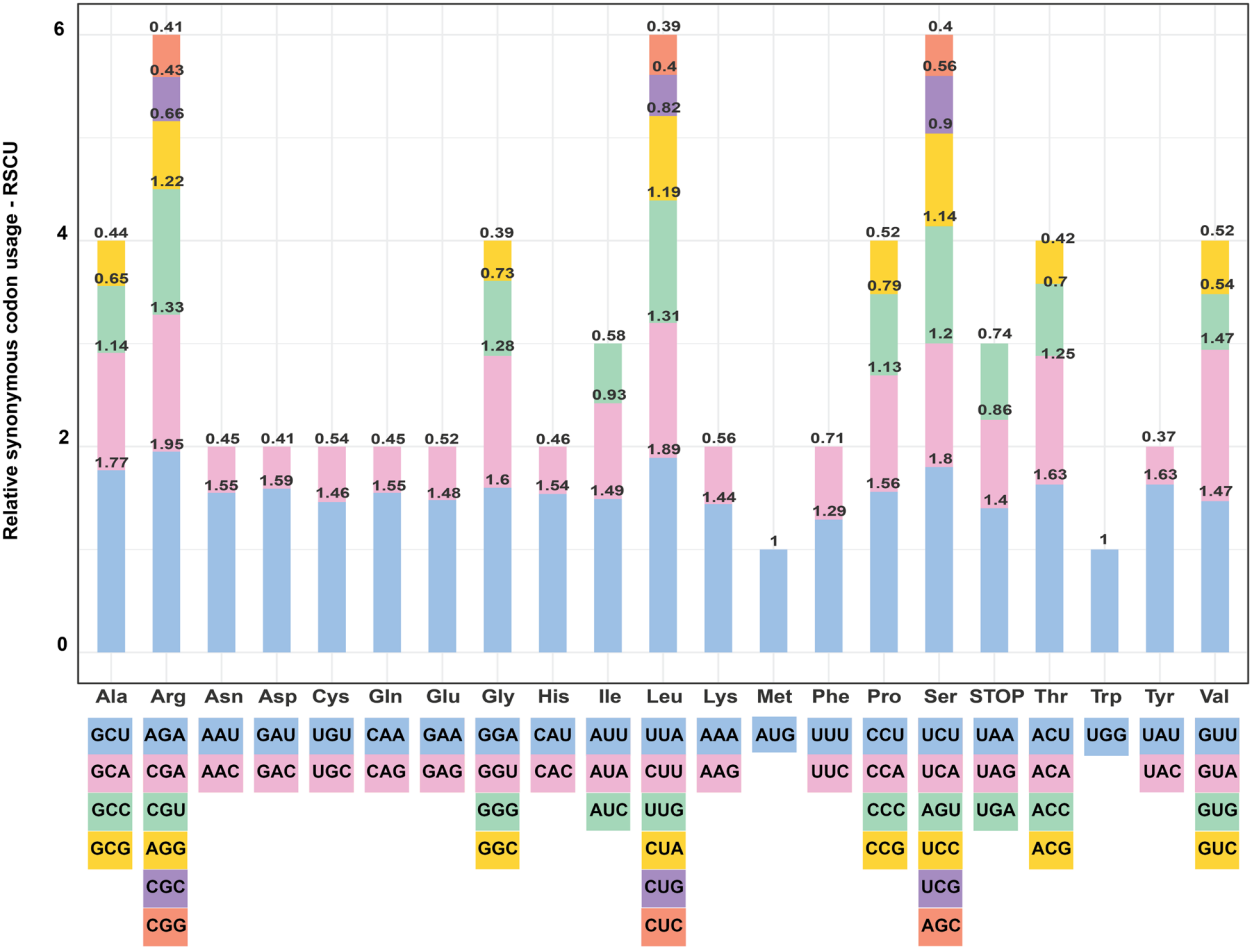
Codon usage bias in protein‐coding genes of *A. banaense*. The y‐axis shows the relative synonymous codon usage (RSCU) values; the x‐axis presents the respective amino acids and codons. Bars, ordered by ascending RSCU values from bottom to top, were colored by codon preference: Light blue, pink, green, yellow, purple, and orange (most to least favored).

The patterns of codon usage in the *A. banaense* cp genome were analyzed by calculating RSCU for 78 PCGs (Figure 8). The results showed a clear bias toward synonymous codons rich in adenine (A) and uracil (U), reflecting the overall AU‐rich composition of the genome. Of the 64 codons, 30 were overrepresented (RSCU > 1), predominantly ending in U or A. Conversely, underrepresented codons (RSCU < 1) were largely composed of cytosine (C) or guanine (G). Methionine and tryptophan were the only amino acids encoded by unique codons, each yielding an RSCU value of one. This preference for AU‐rich codons may be linked to the efficiency of the translational machinery and its adaptation to the nucleotide composition of the genome.

### 3.6 Phylogenetic Analysis

**FIGURE 9:**
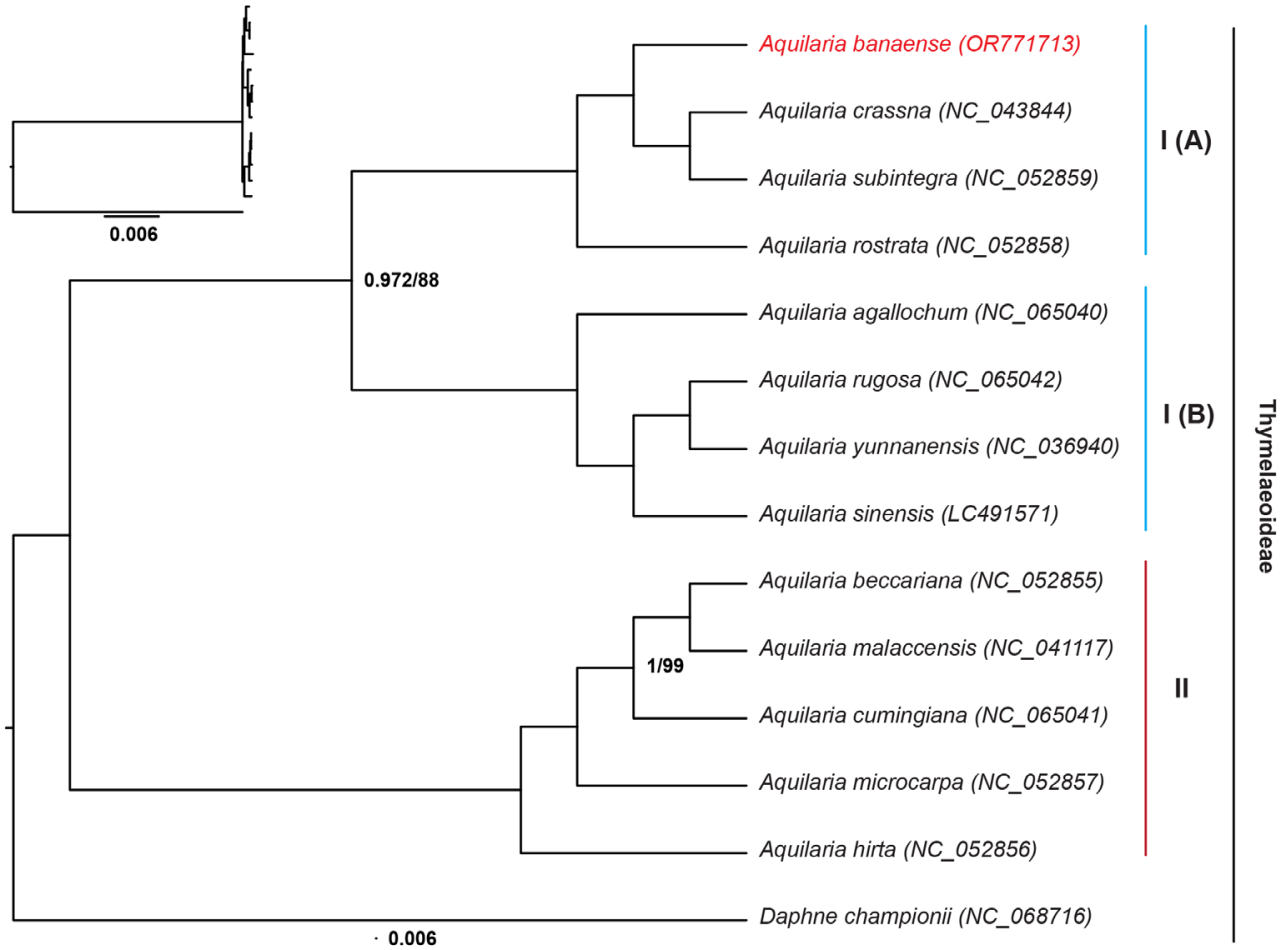
Phylogenetic trees of 13 *Aquilaria* species based on maximum likelihood and Bayesian inference using complete chloroplast genomic data. Unlabeled branches have their posterior probabilities equal to 1 and bootstrap values equal 100. Branches are labeled under the “posterior probability/bootstrap” values format and are drawn to scale proportionally. Tips include species names and sequence accession numbers. A detailed phylogram is inset at the top left. Blue and red lines indicate clades. Sequence references are provided in Table S1.

The phylogenetic relationships among 13 *Aquilaria* species, including the newly sequenced *A. banaense*, were reconstructed using a concatenated alignment of 158,337 cp genome sites. The TVM + I + G substitution model, selected by jModelTest, was applied for both ML and BI analyses. The obtaining ML‐tree and BI‐tree were highly congruent, with strong support from bootstrap values (88) and posterior probabilities (0.972), thus proving high confidence in the phylogenetic results (Figure 9). The genus *Aquilaria* formed a well‐supported monophyletic group, which further resolved into two distinct clades. Clade I includes eight species, which were further subdivided into two robust subgroups, IA and IB, each consisting of four species. Clade II consisted of five species. Notably, *A. banaense* was identified as a sister species to *A. crassna* and *A. subintegra* , and this group clustered together with *A. rugosa* in subgroup IA.

## 4 Discussion

### 4.1 Features of *Aquilaria* Cp Genomes

In this study, we assembled and characterized the complete cp genome of *A*. *banaense*, an endemic agarwood‐producing species native to Vietnam. The genome exhibits the typical quadripartite structure found in most angiosperms, consisting of an LSC region of 87,264 bp, an SSC region of 3342 bp, and two IR regions of 42,102 bp each. Its genome size, GC content, gene composition, and structures align closely with those of other *Aquilaria* species and members of the Thymelaeaceae family, underscoring their evolutionary conservation (Lin et al. 2019; Zhang et al. 2019; Ding et al. 2020; Hishamuddin et al. 2020).

A prominent feature of the *A. banaense* cp genome is the significant expansion of its IRs into the SSC region, which shifts the IR/SSC junction toward the *trnL‐UAG* gene, deviating from the more typical *trnN‐GUU* observed in most land plants (Figure 6). This IR expansion is also observed in other *Aquilaria* species and Thymelaeaceae genera, such as *Daphne*, *Gonystylus*, *Wikstroemia*, *Stellera*, and *Edgeworthia* (Yun et al. 2019; Lee et al. 2020; Liang et al. 2020; He et al. 2021; Qian et al. 2021). The IR regions, constituting nearly half of those genomes, are known for their conservation and slower mutation rates due to gene copy‐correction mechanisms, potentially enhancing genome stability compared to the more variable LSC and SSC regions (Zhu et al. 2016; Weng et al. 2017). The IR expansion likely results from multiple‐step inversions and dispersed repeats, although the precise mechanisms remain uncertain (Wang et al. 2008; Logacheva et al. 2009; Zhu et al. 2016; Ramírez‐Morillo et al. 2024). While this expansion is recurrent in Thymelaeaceae, its evolutionary significance, beyond increasing gene copy number, remains unclear and warrants further investigation.

Regarding gene content, we observed the pseudogenization or complete loss of the *infA* gene in all 13 *Aquilaria* species and the outgroup *Daphne championii* (Table 1). In eight *Aquilaria* cp genomes, *infA* is disrupted by an internal stop codon, rendering it likely non‐functional (Figure S3). Given the essential role of *infA* in initiating protein synthesis within chloroplasts, particularly for the translation of polycistronic mRNAs, its loss could have significant implications for chloroplast function (Hirose et al. 1999). The pseudogenization or loss of *infA* is common in plastid genomes and has been reported in various plant taxa, including *Nicotiana tabacum* , *Arabidopsis thaliana* , and *Oenothera elata* (Shinozaki et al. 1986; Wolfe et al. 1992; Sato et al. 1999; Hupfer et al. 2000; Millen et al. 2001). This gene is frequently lost due to its transfer from the chloroplast to the nuclear genome (Millen et al. 2001; Wicke et al. 2011; AL‐Juhani et al. 2022; Claude et al. 2022; Wu et al. 2023). Whether a similar functional replacement has occurred in *Aquilaria* remains to be determined.

Additionally, all 13 *Aquilaria* species analyzed show the loss of two introns, typically found in the *clpP1* gene, which encodes an ATP‐dependent peptidase essential for plant development. This intron loss is also observed in other Thymelaeaceae genera, such as *Daphne*, *Gyrinops*, and *Wikstroemia* (Chen et al. 2021; Yan et al. 2021; Lee et al. 2022), in contrast to most land plants. Although the functional implications remain unclear, intron loss in *clpP1* has been associated with its elevated substitution rates in lineages such as *Silene*, *Oenothera*, and *Plantago* (Williams et al. 2019). Further sequencing across additional Thymelaeaceae species will be needed to determine whether this intron loss is a synapomorphic feature of the family and to explore the evolutionary trajectory of this gene structure.

### 4.2 Nucleotide Diversity and Potential Molecular Markers

Despite their overall conservation, *Aquilaria* cp genomes exhibit moderate nucleotide diversity (Figure 7). Sliding window analysis using DnaSP v6 identified *rps4‐ndhJ* and *ndhF‐rpl32* as the most variable regions across the 13 *Aquilaria* species. Interestingly, *ycf1*, a commonly variable region in angiosperms (Dong et al. 2012; Amar 2020), exhibited a low level of divergence (Pi‐value = 0.001), likely due to its relocation to the highly conserved IR regions. Additionally, *A. banaense* showed notable variation in the *accD* gene compared to other *Aquilaria* species (Figure 5).

These identified divergent regions offer potential markers for species identification, critical for the conservation and trade regulation of this endangered genus. To utilize these regions effectively, future studies could focus on designing PCR primers that target conserved flanking sequences to allow for reliable amplification across different *Aquilaria* species. The resulting PCR products can then be sequenced to identify species‐specific polymorphisms. Such markers would be invaluable for conservation efforts, particularly in combating the illegal trade of agarwood.

### 4.3 Phylogenetic Placement and Future Directions

Phylogenetic analyses of cp genomes have significantly advanced our understanding of *Aquilaria* taxonomy and evolution, as shown by prior studies (Ding et al. 2020; Hishamuddin et al. 2020; Lee et al. 2022; Kan et al. 2024). Our phylogenetic results aligned with these studies, revealing distinct groupings within *Aquilaria* (Figure 9). Group IA includes *A. banaense* (central Vietnam: Quang Nam, Thua Thien‐Hue), a sister species to *A. crassna* (Cambodia, Laos, southern Vietnam) and *A. subintegra* (Thailand), clustering with *A. rostrata* (Peninsular Malaysia). Group IB comprises species *A. agallochum* (Northeast India, Bhutan, and Myanmar), *A. rugosa* (northern Vietnam, Thailand) (Le et al. 2005), *A. yunnanensis* (Yunnan, China; northern Vietnam), and *A. sinensis* (southern China). These subgroups (IA and IB) form a sister clade to group II, which includes *A. beccariana* (Sumatra, Borneo), *A. cumingiana* (Philippines), *A. hirta* (Sumatra, Java, and Malaysia), *A. malaccensis* (widespread but primarily insular: Sumatra, Borneo, and Malaysia), and *A. microcarpa* (Sumatra and Borneo) (Lee and Mohamed 2016) (Table S1).

Our chloroplast genome analysis firmly places *A. banaense* within a mainland Southeast Asian clade, where it is sister to *A. crassna* and *A. subintegra* . This phylogenetic structure shows a potential correlation with geography, separating mainland species (Clade I) from insular ones (Clade II), as also discussed by others (Hishamuddin et al. 2020; Lee et al. 2022). However, we recognize that a comprehensive understanding of the genus's deep biogeographical history and evolutionary trajectories will require expanded genomic data from a wider array of species and populations, especially from underrepresented regions like Malesia.

Despite this limitation, our results provide a useful framework for conservation, particularly within the legal context of Vietnam. In Vietnam, the harvesting and trade of all wild agarwood‐producing species are prohibited; only planted specimens of *A. crassna* are permitted for trade. Given that *A. banaense* is an endemic species often found in the same regions, the ability to accurately distinguish it from *A. crassna* is paramount. The divergent regions identified (Section 4.2) can be used to create reliable markers that correctly identify *A. banaense*, thereby helping to prevent illegal trading and the poaching of this protected endemic species. Moreover, these same markers may be used to investigate population structure, genetic diversity, gene flow, and demographic history in future studies of *A. banaense*, which are critical for assessing population viability and developing targeted conservation strategies.

In conclusion, this study provides a critical cp genomic resource that clarifies the evolutionary position of *A. banaense*. It not only reinforces its conservation importance but also delivers the foundational data needed for tangible conservation actions, from preventing illegal trade to designing long‐term species management plans.

## Author Contributions

**Yen Thi Van:** conceptualization (equal), formal analysis (equal), funding acquisition (equal), investigation (equal), resources (equal), writing – review and editing (equal). **Ngoc Bao Mach:** formal analysis (equal), investigation (equal), methodology (equal), visualization (equal), writing – original draft (equal), writing – review and editing (equal). **Thanh‐Thuy Duong:** conceptualization (equal), funding acquisition (equal), investigation (equal), resources (equal), supervision (equal), writing – review and editing (equal). **Thang Nam Tran:** investigation (supporting), validation (equal). **Nguyen Van Minh:** investigation (supporting), validation (equal). **Tan Duy Ngoc Nguyen:** investigation (supporting), validation (equal). **Hoang Dang Khoa Do:** investigation (equal), methodology (equal), resources (equal), validation (equal), writing – review and editing (equal). **Thiet Minh Vu:** conceptualization (equal), methodology (equal), resources (equal), visualization (equal), writing – original draft (equal), writing – review and editing (lead).

## Conflicts of Interest

The authors declare no conflicts of interest.

## Supporting information


**FIGURE S1.**Sequencing depth and coverage of the *A. banaense* cp genome. The x‐axis represents the genomic position, and the y‐axis shows the sequencing depth.
**FIGURE S2.** Structures of *cis*‐ and *trans*‐splicing genes in the *A. banaense* cp genome. The diagram illustrates the organization of the *trans*‐splicing gene *rps12* (top) and 11 *cis*‐splicing genes (bottom). For *rps12*, the only *trans*‐splicing gene, two transcripts are shown, with exons colored by their genomic regions (LSC, IRA, IRB). Exons 2 and 3 are duplicated in the IR regions, resulting in two copies each. For *cis*‐splicing genes, structures are depicted with exons (black blocks) and introns (white blocks). *ndhA* and *ndhB* each have two copies due to IR duplication. Genomic positions (bp) are labeled, and arrows indicate transcription direction.
**FIGURE S3.** MAFFT alignment of the *infA* gene structure in *Aquilaria* species compared to *Hibiscus cannabinus* (GenBank ID NC_045873). Numbers indicate gene length (bp). Amino acids use single‐letter codes; stop codons are marked with an asterisk (*). Dashes represent alignment gaps. Colors highlight nucleotide variations.
**TABLE S1.** Summary of species included in phylogenetic analysis and their distribution. SEA is Southeast Asia.

## Data Availability

The assembled genome sequences and raw sequencing data (SRA) are accessible in the NCBI GenBank database under the accession numbers OR771713 and PRJNA1029340, respectively, with BioSample accession SAMN37865748.
