# Supplementary material for: The Complete Chloroplast Genome of the Vietnamese Endemic Species Aquilaria banaense P.H. Hô, 1986 (Thymelaeaceae): Structure, Evolution, and Phylogeny
Source: Ecol Evol. 2025 Jul 27;15(7):e71708. doi: 10.1002/ece3.71708 (PMC12301069; doi:10.1002/ece3.71708)
Supplement: Supplementary file 1 — FIGURE S1.Sequencing depth and coverage of the A. banaense cp genome. The x‐axis represents the genomic position, and the y‐axis shows the sequencing depth. FIGURE S2. Structures of cis‐ and trans‐splicing genes in the A. banaense cp genome. The diagram illustrates the organization of the trans‐splicing gene rps12 (top) and 11 cis‐splicing genes (bottom). For rps12, the only trans‐splicing gene, two transcripts are shown, with exons colored by their genomic regions (LSC, IRA, IRB). Exons 2 and 3 are duplicated in the IR regions, resulting in two copies each. For cis‐splicing genes, structures are depicted with exons (black blocks) and introns (white blocks). ndhA and ndhB each have two copies due to IR duplication. Genomic positions (bp) are labeled, and arrows indicate transcription direction. FIGURE S3. MAFFT alignment of the infA gene structure in Aquilaria species compared to Hibiscus cannabinus (GenBank ID NC_045873). Numbers indicate gene length (bp). Amino acids use single‐letter codes; stop codons are marked with an asterisk (*). Dashes represent alignment gaps. Colors highlight nucleotide variations. TABLE S1. Summary of species included in phylogenetic analysis and their distribution. SEA is Southeast Asia. [file ECE3-15-e71708-s001.docx]

Supporting information for the manuscript

Title:

**The complete chloroplast genome of the Vietnamese endemic species *Aquilaria banaense* P.H.Hô, 1986 (Thymelaeaceae): Structure, Evolution, and Phylogeny**

*Yen Thi Van^1*^, Ngoc Bao Mach^2*^, Thanh- Thuy Duong^1^*,* *Thang Nam Tran^1^, Minh Van Nguyen^1^, Tan Duy Ngoc Nguyen^1^*

*Hoang Dang Khoa Do^2,^, Thiet Minh Vu^2,3^*

^1^University of Agriculture and Forestry, Hue University, Hue City, Vietnam, 49000

^2^Functional Genomic Research Center, NTT Hi-Tech Institute, Nguyen Tat Thanh University, Ho Chi Minh City, Vietnam, 70000

^3^Nguyen Tat Thanh University Center for High Technology Development, Saigon Hi-Tech Park, Ho Chi Minh City, Vietnam, 70000

*Yen Thi Van, Ngoc Mach Bao, and Thanh Thuy Duong should be considered joint first author

Corresponding authors: Yen Thi Van, email: vanthiyen@hueni.edu.vn; Thiet Minh Vu, email: [vmthiet@ntt.edu.vn](mailto:vmthiet@ntt.edu.vn)


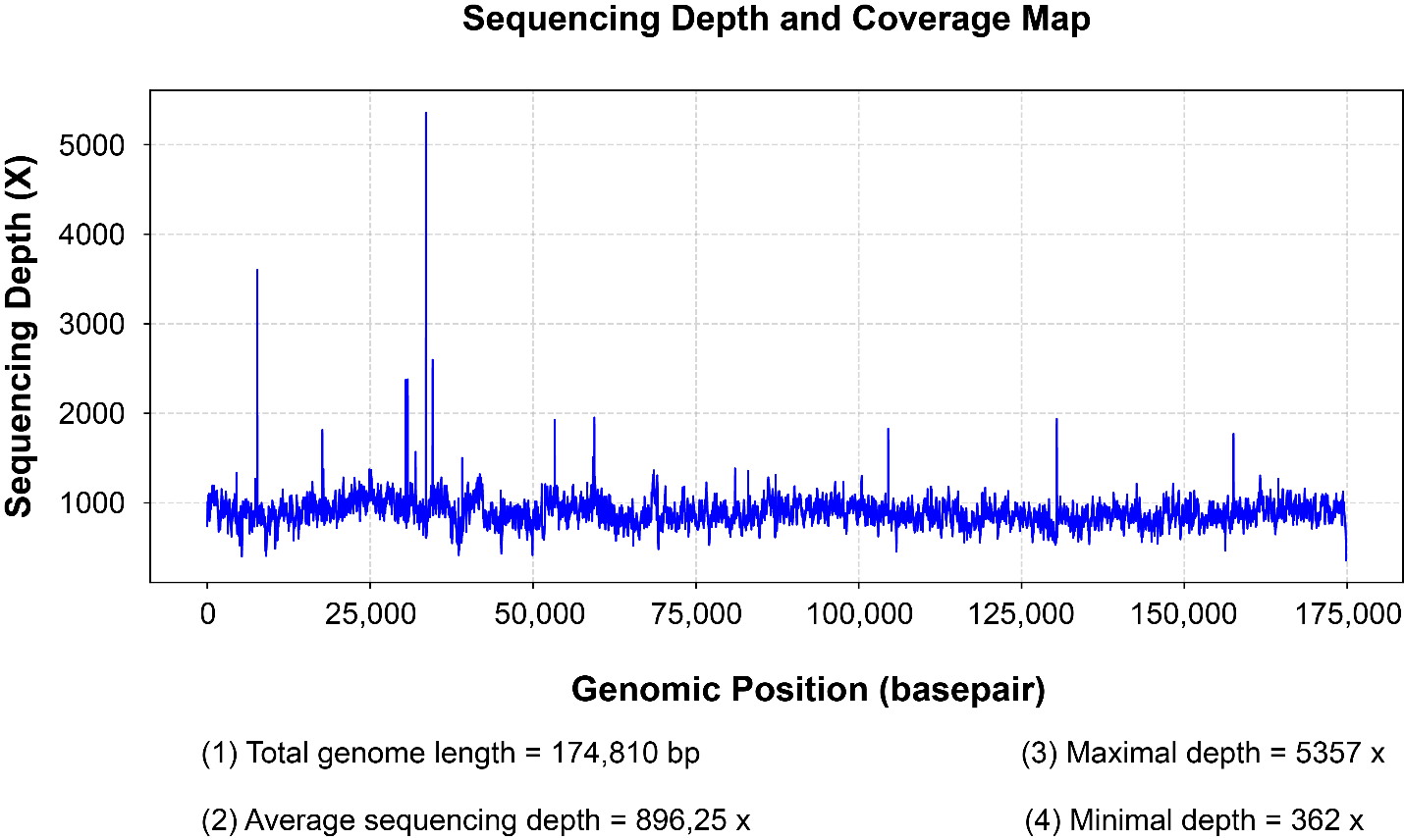


FIGURE S1. Sequencing coverage of the *Aquilaria banaense* chloroplast genome.

**TABLE S1**. Summary of species included in phylogenetic analysis and their distribution. SEA is SouthEast Asia

| No. | Species | GenBank ID | Cp genome references | Geographical distribution | Endemic Status | Phylogenetic Group |
| --- | --- | --- | --- | --- | --- | --- |
| 1 | *Aquilaria agallochum* | NC_065040 | (Lee *et al.*, 2022) | Northeast India, Bhutan, Bangladesh, Myanmar | Not endemic | IB (South China, South Asia) |
| 2 | *Aquilaria banaense* | OR771713 | This study | Central Vietnam | Endemic (Vietnam) | IA (Mainland SEA) |
| 3 | *Aquilaria beccariana* | NC_052855 | (Hishamuddin *et al.*, 2020) | Malaysia and Indonesia (Sumatra, Borneo) | Not endemic | Insular SEA |
| 4 | *Aquilaria crassna* | NC_043844 | (Li *et al.*, 2019) | Cambodia, Laos, Southern Vietnam, Thailand | Not endemic | IA (Mainland SEA) |
| 5 | *Aquilaria cumingiana* | NC_065041 | (Lee *et al.*, 2022) | Philippines (Mindanao, Leyte) | Endemic (Philippines) | Insular SEA |
| 6 | *Aquilaria hirta* | NC_052856 | (Hishamuddin *et al.*, 2020) | Malaysia and Indonesia (Sumatra, Java) | Not endemic | Insular SEA |
| 7 | *Aquilaria malaccensis* | NC_041117 | (Lee *et al.*, 2018) | India, Bangladesh, Bhutan, Indonesia, Malaysia, Myanmar, Thailand | Not endemic | Insular SEA |
| 8 | *Aquilaria microcarpa* | NC_052857 | (Hishamuddin *et al.*, 2020) | Indonesia (Sumatra, Borneo), Malaysia | Not endemic | Insular SEA |
| 9 | *Aquilaria rostrata* | NC_052858 | (Hishamuddin *et al.*, 2020) | Peninsular Malaysia | Not endemic? | IA (Mainland SEA) |
| 10 | *Aquilaria rugosa* | NC_065042 | (Lee *et al.*, 2022) | Northern Vietnam, Southern China, Laos | Not endemic | IB (South China, South Asia) |
| 11 | *Aquilaria sinensis* | LC491571 | (Lin *et al.*, 2019) | Southern China (Guangdong, Guangxi, Hainan) | Endemic (China) | IB (South China, South Asia) |
| 12 | *Aquilaria subintegra* | NC_052859 | (Hishamuddin *et al.*, 2020) | Thailand | Endemic (Thailand) | IA (Mainland SEA) |
| 13 | *Aquilaria yunnanensis* | NC_036940 | (Zhang *et al.*, 2019) | China (Yunnan), Northern Vietnam | Mostly endemic (China, extends to Vietnam) | IB (South China, South Asia) |
| 14 | *Daphne championii* | NC_068716 | Direct submission |  |  |  |


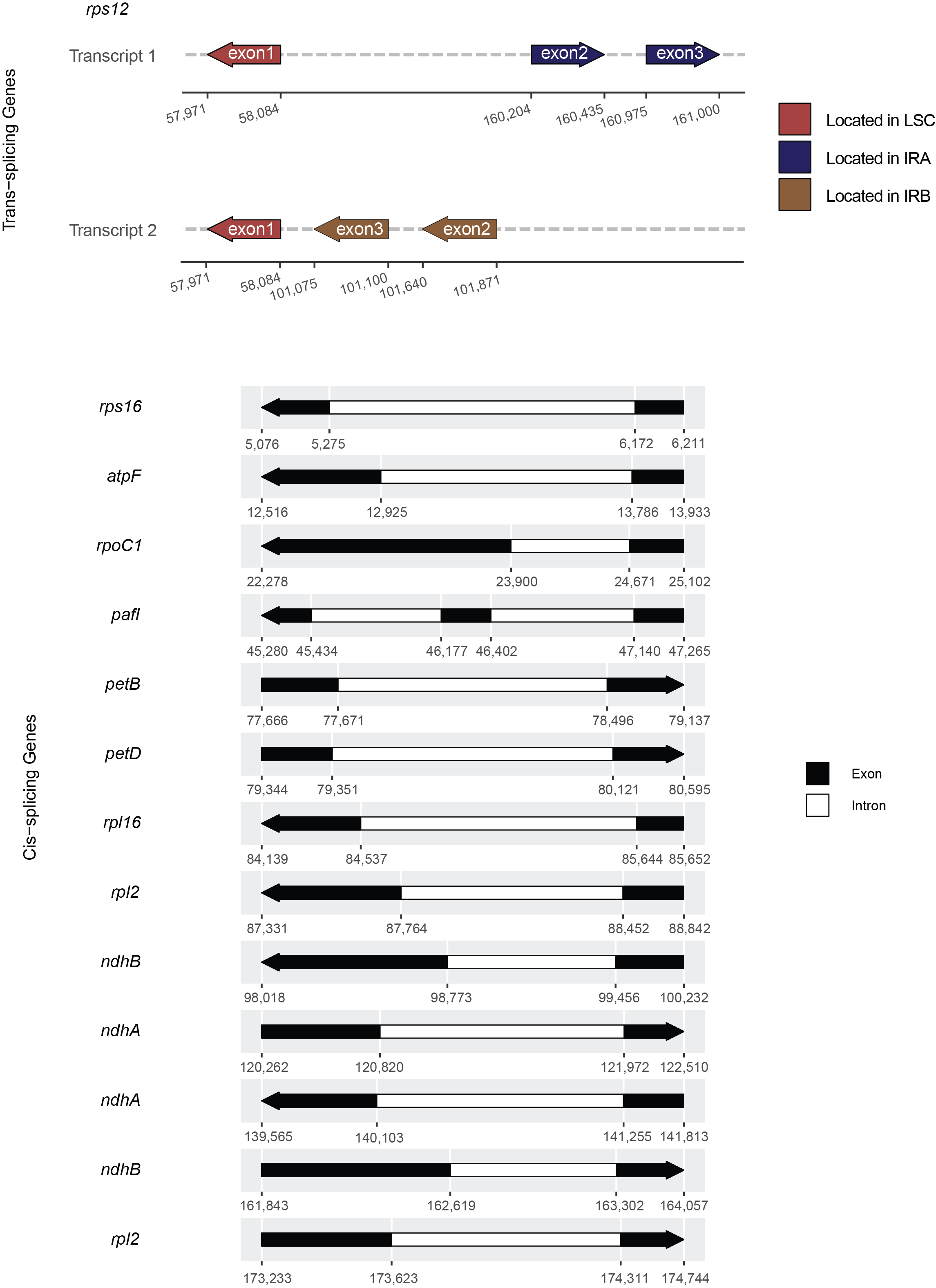


**FIGURE S2**. Structures of *cis-* and *trans-*splicing genes in the *A. banaense* cp genome. The diagram illustrates the organization of the *trans*-splicing gene *rps12* (top) and 11 *cis*-splicing genes (bottom). For *rps12*, the only *trans*-splicing gene, two transcripts are shown, with exons colored by their genomic regions (LSC, IRA, IRB). Exons 2 and 3 are duplicated in the IR regions, resulting in two copies each. For *cis*-splicing genes, structures are depicted with exons (black blocks) and introns (white blocks). *ndhA* and *ndhB* each have two copies due to IR duplication. Genomic positions (bp) are labeled, and arrows indicate transcription direction.


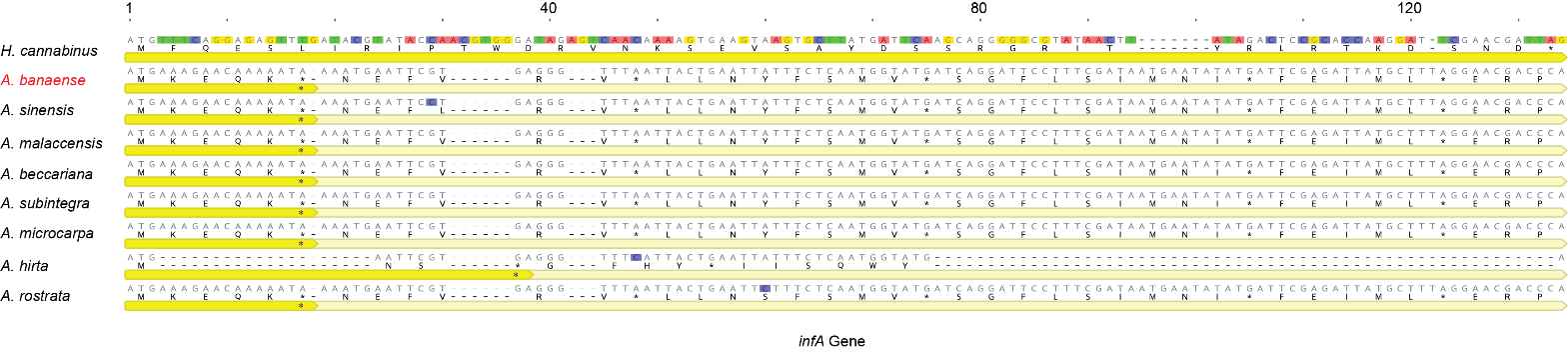


FIGURE S3. MAFFT alignment of the *infA* gene structure in *Aquilaria* species compared to *Hibiscus cannabinus* (GenBank ID NC_045873). Numbers indicate gene length (bp). Amino acids use single-letter codes; stop codons are marked with an asterisk (*). Dashes represent alignment gaps. Nucleotide variations are highlighted by colors.

REFERENCES

Hishamuddin, M.S. *et al.* (2020) ‘Comparison of eight complete chloroplast genomes of the endangered *Aquilaria* tree species (Thymelaeaceae) and their phylogenetic relationships’, *Scientific Reports*, 10(1), p. 13034. Available at: https://doi.org/10.1038/s41598-020-70030-0.

Lee, S.Y. *et al.* (2018) ‘The complete chloroplast genome of *Aquilaria malaccensis* Lam. (Thymelaeaceae), an important and threatened agarwood-producing tree species’, *Mitochondrial DNA Part B*, 3(2), pp. 1120–1121. Available at: https://doi.org/https://doi.org/10.1080/23802359.2018.1519382.

Lee, S.Y.I.H. *et al.* (2022) ‘Phylogenetic relationships of *Aquilaria* and *Gyrinops* (Thymelaeaceae) revisited: evidence from complete plastid genomes’, *Botanical Journal of the Linnean Society*, 200(3), pp. 344–359. Available at: https://doi.org/10.1093/BOTLINNEAN/BOAC014.

Li, G.-D. *et al.* (2019) ‘The complete chloroplast genome of a critically endangered agarwood tree, *Aquilaria crassna* (Thymelaeaceae)’, *Mitochondrial DNA Part B*, 4(1), pp. 1810–1811. Available at: https://doi.org/10.1080/23802359.2019.1613200.

Lin, C.P. *et al.* (2019) ‘The complete chloroplast genome of agarwood producing species, *Aquilaria sinensis* (Lour.) Gilg: a species on IUCN red list’, *Mitochondrial DNA Part B*, 4(2), pp. 2992–2993. Available at: https://doi.org/10.1080/23802359.2019.1664954.

Zhang, Y.H. *et al.* (2019) ‘Characterization of the complete chloroplast genome of the vulnerable agarwood tree, *Aquilaria yunnanensis* (Thymelaeaceae)’, *Conservation Genetics Resources*, 11(2), pp. 161–164. Available at: https://doi.org/10.1007/S12686-018-0989-0.
